# Association of bone mineral density and depression in different bone sites and ages: A meta‐analysis

**DOI:** 10.1002/fsn3.2379

**Published:** 2021-07-19

**Authors:** Shiyi Yuan, Jianjun Chen, Li Zeng, Chanjuan Zhou, Shenrun Yu, Liang Fang

**Affiliations:** ^1^ Department of Nephrology Yongchuan Hospital of Chongqing Medical University Chongqing China; ^2^ Department of Nephrology The People’s Hospital of Yongchuan District Chongqing China; ^3^ College of Life Sciences Chongqing Medical University Chongqing China; ^4^ Department of Neurology The Second Affiliated Hospital of Chongqing Medical University Chongqing China; ^5^ The People’s Hospital of Yong chuan District Chongqing China

**Keywords:** bone mineral density, depression, meta‐analysis

## Abstract

Major depressive disorder (MDD) is considered as a risk factor for osteoporosis. Bone mineral density (BMD), as the main tool for diagnosing osteoporosis, has been reported to have correlation with MDD in different cohorts. However, the information in causative link and etiology determinants of osteoporosis in MDD is still under investigation. The results are unclear. Thus, we perform a meta‐analysis to evaluate the association between altered BMD and MDD. We searched the electronic databases to find studies examining BMD in patients with MDD. Finally, 26 published studies were included in our meta‐analysis up from January 1990 to January 2019. All the data were pooled analysis using RevMan software. The association between altered BMD and MDD was assessed by std. mean difference (STD) and their 95% confidence intervals (CIs) for each study. Twenty‐six studies were included in this meta‐analysis. Pooled results showed a significant lower BMD in spine (STD=0.51, 95% CI=0.30–0.71, *p* < .00001), total hip (STD=0.41, 95% CI=0.16 to 0.66, *p* = .001), and femoral neck (STD=0.93, 95% CI=0.32 to 1.55, *p* = .003) in MDD compared with controls. After stratification by mean age, gender, recruitment, diagnostic criteria, and measuring methods, no significant difference of BMD was found in bone mineral density of male total hip between MDD and controls(*p* > .05). Moreover, adults appear to have lower BMD than old cohorts. This is an updated meta‐analysis to reveal the a**ssociation of bone mineral density and depression**, suggesting that BMD appears to be more susceptible to occur in spine, total hip, femoral neck in MDD, especially for adults and women. Our meta‐analysis may provide clinicians and public health administrators with an important screening tool for assessing depression and avoiding osteoporosis in adult subjects and female.

## Graphical abstract

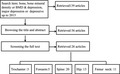

## 1 INTRODUCTION

Major depression disorder (MDD) is a kind of mental illness. The typical manifestation is persistent depression and loss of interest (Boku and Nakagawa 2018). According to clinical and animal model trials, converged lines of evidence suggested that dysfunction of hippocampal neurogenesis (Kleschevnikov and Belichenko 2012), immune system (Tesch, 2017), hypothalamic–pituitary–adrenal (HPA) axis (Dalfsen and Markus, 2018), and host microbiome metabolism (Pak and Cummings 2019) were related to the pathophysiological mechanisms of MDD.

Additionally, several studies have reported that brain‐to‐bone signal was considered to be a link between MDD and osteoporosis (Jones et al. 2004), suggesting there is a relationship between MDD and osteoporosis. Bone mineral density (BMD) determination was currently the main tool for diagnosing osteoporosis. In particular, previous studies have found the association between depression and lower BMD ever since the first prospective case–control design by Schweiger et al. (1994) and several studies followed up with findings alike to Schweiger's work. While, negative associations have also been identified in different cohorts. Since a variety of pathophysiological mechanisms have been shown to cause low BMD, including post‐menopausal condition, physical activity, and age, the discrepancy was possibly limited by significant shortcomings such as sample size, measuring methods, age, study design, and inclusion criteria. Accordingly, we carried out an updated meta‐analysis to evaluate the association between depression and osteoporosis and to find out the possible causative factors.

## 2 META‐ANALYSIS METHODS

### 2.1 Search strategy

Several electronic databases (EMBASE, Google Scholar, Science Direct, Springer, PubMed) were searched systematically to identify all the published studies about the association between BMD and/or osteoporosis and MDD from January 1990 to January 2019 with those key words: (“osteoporosis” OR “bone mineral density” OR “BMD” OR “bone”) AND (“depression” OR “major depressive disorder” OR “depressive episode” OR “MDD” OR “depression”), and relevant Medical Subject Heading (MeSH) terms were utilized. The reference lists of all articles were also hand‐searched.

### 2.2 Inclusion and exclusion criteria

Inclusion criteria were as follows: (i) a clinical case–control study, including population‐based study; (ii) measuring the BMD in MDD and control cohorts; (iii) the diagnostic criteria of the patients were introduced in detail; (iv) sufficiently reported data for assessing std. mean difference (SMD) and the 95% confidence intervals (95% CIs); and (v) full‐length published articles. Conference papers, follow‐up designs, abstracts, case‐report studies, reviews were excluded.

### 2.3 Quality assessment

Two investigators separately rated the quality of the retrieved studies. Study quality was assessed using Newcastle–Ottawa Quality Assessment Scale.

### 2.4 Data extraction and collection

Two authors (LZ and SYY) independently obtained data to avoid extraction bias and discussed the differences to reach agreement. Those information was recorded from each eligible article, including first author, country of origin, publication year, mean age, number of cases and controls (female/male), BMD (expressed in g/cm^2^), measuring methods, measuring outcome or index, diagnostic criteria for subjects, and measuring bone site information.

### 2.5 Statistical methods

The difference in BMD between MDD and controls at five most commonly measured bone sites was analyzed, including spine, total hip, femoral neck, femoral trochanter, and forearm. All data analyses were carried out by Rev Man 5.0.1. The association between BMD and MDD was assessed by estimating SMD and 95% CIs Greater weight was commonly considered to be a study of larger samples and higher quality; this procedure corrected the biases associated with small sample sizes. Statistical heterogeneity across studies was expressed by the I^2^ tests (Higgins J P, Thompson S G. Quantifying heterogeneity in a meta‐analysis.[J]. Statistics in Medicine, 2002, 21(11):1539.). Studies with an I^2^≥50% were considered that the degree of heterogeneity was insignificant; I^2^<50% was considered to have significant heterogeneity, respectively (Higgins J P T, Thompson S G, Deeks J J, et al. Measuring inconsistency in meta‐analyses. Bmj, 2003, 327(7,414):557–560.). *p* <.05 was considered significantly different. For subgroup analysis, we also compared studies based on diagnosis of depression, mean age, and gender (female/male) and used samples. In order to evaluate the possible bias, sensitivity analysis was carried out by deleting individual studies consecutively to try to evaluate the contribution of each individual dataset to the set SMD. Therefore, publication bias and the tendency of large effect in small studies were assessed by Begg's funnel plots while asymmetry of funnel plot suggested bias existing.

## 3 RESULTS

### 3.1 Literature search results

**FIGURE 1:**
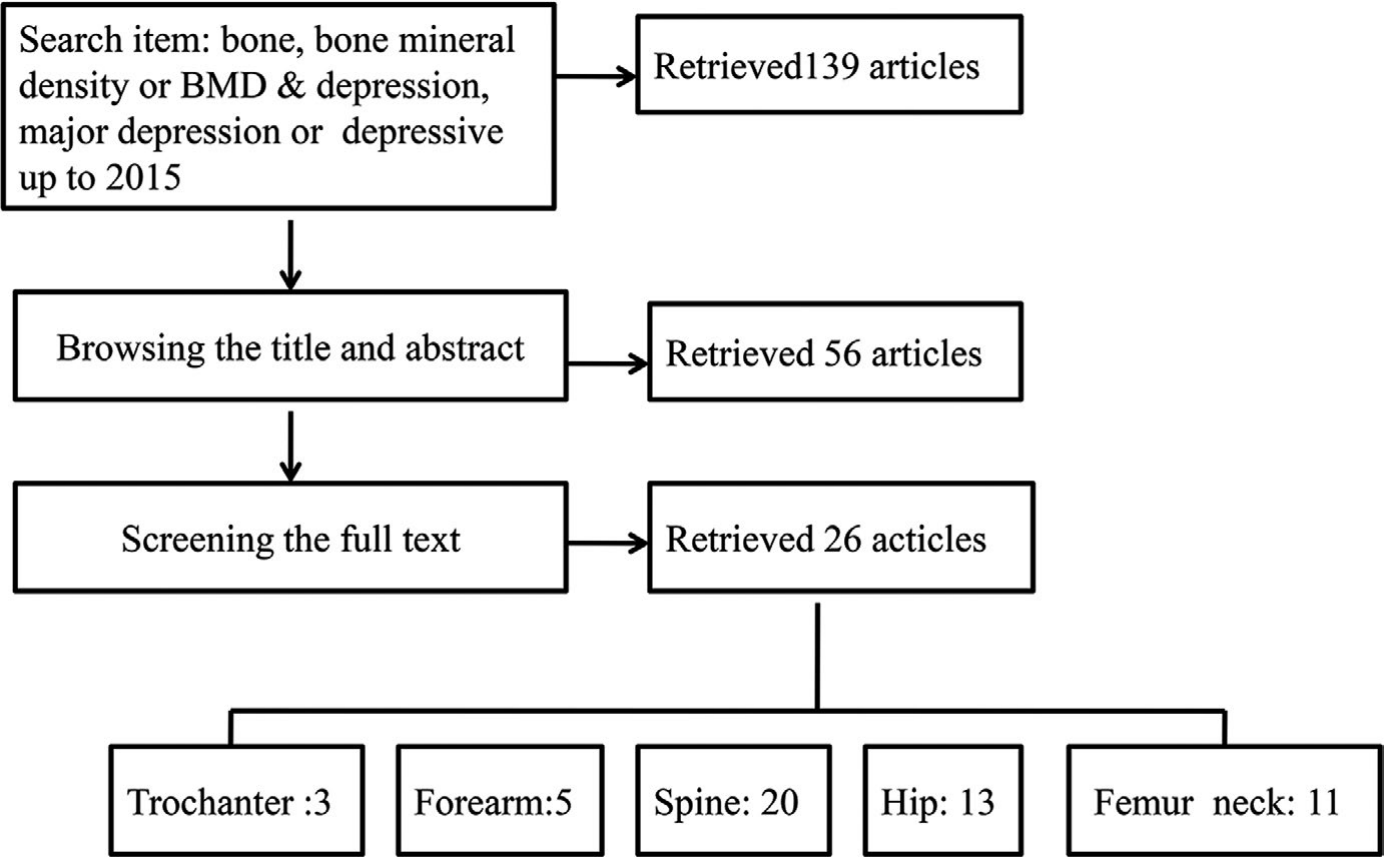
Workflow of meta‐analysis

The procedure is shown in Figure 1. There were 139 studies involving potentially relevant published data, and 56 were retained after screening titles and abstracts. And 29 studies were excluded due to those reasons: (i) 9 studies were reviews about depression and osteoporosis or BMD (Bab & Yirmiya, 2010; Carlone et al. 2015; Cizza et al. 2009; Gold & Solimeo, 2006; Ilias et al. 2006; Williams et al. 2009); (ii) 7 studies assessed antidepressant medications and osteoporosis (Diem, Blackwell, Stone, Yaffe, Haney, et al., 2007; Haney et al. 2007; Williams et al. 2008; Aydin et al. 2011; Rizzoli et al. 2012; Diem et al. 2013; Bruyère & Reginster, 2014); (iii) 3 studies were not a case–control design (Coelho et al. 1999; Jacka et al. 2005; Lunsford et al. 2014); (iv) 1 study did not measure BMD levels (Tolea et al. 2007); (v) 5 studies reported osteoporosis with normalized BMD value or T‐score or Z‐score without raw data (Erez et al. 2012; Furlan et al. 2005; Govender et al. 2010; Kurmanji et al. 2010; Lourenço et al. 2014); (vi) 4 studies were meta‐analyses up to 2009 (Cizza et al. 2010; Wu et al., 2009, 2010; Yirmiya & Bab, 2009); and (vii) 1 study was a follow‐up study using duplicated population (Schweiger et al. 2000). Finally, there were 26 studies included in our meta‐analysis from January 1990 to January 2019 (Schweiger et al. 1994; Michelson et al. 1996; Amsterdam & Hooper, 1998; Reginster et al. 1999; Whooley et al. 1999; Schweiger et al. 2000; Robbins et al., 2001; Kavuncu et al. 2002; Yazıcı et al. 2003; Mussolino et al. 2004; Whooley et al. 2004; Ozsoy et al. 2005; Søgaard et al. 2005; Wong et al. 2005; Yazıcı et al. 2005; Kahl et al. 2006; Altindag et al. 2007; Diem, Blackwell, Stone, Yaffe, Cauley, et al., 2007; Eskandari et al., 2007; Mezuk et al. 2008; Petronijević et al. 2008; Williams et al. 2011; Atteritano et al. 2013; Fazeli et al. 2013; Calarge et al. 2014; Rauma et al. 2015). Table 1 describes the primary characteristics of the eligible studies in more detail.

**TABLE 1:** Key characteristics of included studies from 1990

Study	Year	Country	Group	Mean age(*SD*)	Number	Bone site and BMD(g/cm^2^)				
						Spine	Femoral neck	Forearm	Total hip	Trochanter
Schweiger	1994	Germany	no depression	60(12)	57	1.01 ± 0.41	*N*/A	*N*/A	*N*/A	*N*/A
			depression	60.5(10.5)	80	0.91 ± 0.43				
Michelson	1996	USA	no depression	41(7)	24	0.93 ± 0.08	0.88 ± 0.11	*N*/A	*N*/A	0.74 ± 0.08
			depression	41(8)	24	0.87 ± 0.12	0.76 ± 0.11			0.66 ± 0.11
Amsterdam	1998	USA	no depression	37.8(3.6)	5	1.176 ± 0.01	*N*/A	*N*/A	*N*/A	*N*/A
			depression	41.3(12.8)	6	1.166 ± 0.01				
Reginster	1999	Belgium	no depression	——	12	0.921 ± 0.01	0.710 ± 0.01	*N*/A	0.821 ± 0.01	*N*/A
			depression	——	12	0.905 ± 0.02	0.677 ± 0.01		0.776 ± 0.01	
Whooley	1999	USA	no depression	73.3(5.1)	6,895	0.86 ± 0.17	*N*/A	*N*/A	0.76 ± 0.13	*N*/A
			depression	74.5(5.3)	461	0.85 ± 0.17			0.76 ± 0.13	
Schweiger	2000	Germany	no depression	64(10)	21	0.97 ± 0.51	*N*/A	*N*/A	*N*/A	*N*/A
			depression	59(11)	18	0.88 ± 0.34				
Robbins	2001	USA	no depression	74.21 (4.61)	1,319	*N*/A	*N*/A	*N*/A	0.83 ± 0.18	*N*/A
			depression	74.87 (5.56)	230				0.79 ± 0.18	
Kavunco	2002	Turkey	No depression	36.7(6.7)	42	1.160 ± 0.128	0.997 ± 0.121	*N*/A	1.038 ± 0.106	0.854 ± 0.10
			depression	35.4(7.5)	42	1.163 ± 0.123	0.984 ± 0.112		1.024 ± 0.122	0.847 ± 0.114
Yazıcı	2003	Turkey	no depression	31.2(7.9)	15	1.108 ± 0.085	0.859 ± 0.118	*N*/A	0.953 ± 0.086	1.095 ± 0.126
			depression	30.8(8.4)	25	0.978 ± 0.143	0.768 ± 0.112		0.851 ± 0.13	0.989 ± 0.152
Mussolino	2004	USA	no depression	29.8	4,747	*N*/A	*N*/A	*N*/A	1.001	*N*/A
			depression	30.3	424				0.976	
Whooley	2004	USA	no depression	66.7 ( 7.5)	497	1.08 ± 0.17	*N*/A	*N*/A	0.95 ± 0.14	*N*/A
			depression	64.6 ( 8.6)	16	1.07 ± 0.17			0.93 ± 0.14	
Ozsoy	2005	Turkey	no depression	33.73 ± 7.16	23	0.99 ± 0.09	0.82 ± 0.12	*N*/A	*N*/A	*N*/A
			depression	37.57 ± 8.70	42	0.96 ± 0.13	0.86 ± 0.20			
Sogaard	2005	Norway	no depression	40.5	1,437	*N*/A	*N*/A	0.552 ± 0.2	*N*/A	*N*/A
			depression	40.7	343			0.536 ± 0.2		
Wong	2005	Hongkong	no depression	72.34 ± 4.96	1,830	0.95 ± 0.18	*N*/A	*N*/A	0.87 ± 0.13	*N*/A
			depression	72.94 ± 5.41	169	0.94 ± 0.20			0.83 ± 0.13	
Yazıcı	2005	Turkey	no depression	46.2 ± 4.2	30	0.937 ± 0.40	0.745 ± 0.511	*N*/A	*N*/A	*N*/A
			depression	44.8 ± 5.4	35	1.021 ± 0.07	0.883 ± 0.13			
Kahl	2006	Germany	no depression	18–43	16	1.25 ± 0.03	1.05 ± 0.03	0.52 ± 0.01	*N*/A	*N*/A
			depression	20–51	23	1.21 ± 0.05	1.05 ± 0.04	0.56 ± 0.02		
Altindag	2007	Turkey	no depression	42.8 ± 5.3 (26–56)	41	98.9 ± 2.5	108.0 ± 2.2	*N*/A	*N*/A	
			depression	39.8 ± 8.8 (33–54)	36	94.7 ± 3.2	103.9 ± 2.8			
Diem	2007	USA	no depression	75.6 ± 4.1	3,977	*N*/A	*N*/A	*N*/A	0.7 ± 0.1	*N*/A
			depression	76.7 ± 4.3	200				0.7 ± 0.1	
Eskandari	2007	USA	no depression	35 ± 6.8	44	1.043 ± 0.092	0.866 ± 0.094	*N*/A	0.973 ± 0.104	*N*/A
			depression	35 ± 6.9	89	1.02 ± 0.12	0.849 ± 0.121		0.963 ± 0.120	
Petronijević	2007	Serbia	no depression	40.5 ± 5.7	47	1.218 ± 0.118	1.003 ± 0.090	*N*/A	*N*/A	*N*/A
			depression	40.7 ± 4.6	73	1.007 ± 0.132	0.821 ± 0.120			
Mezuk	2008	USA	no depression	——	83	1.19	*N*/A	*N*/A	*N*/A	*N*/A
			depression	——	10	1				
Williams	2011	Australia	no depression	66.0 (47.0–73.0)	6,290	*N*/A	*N*/A	0.385 ± 0.081	*N*/A	*N*/A
			depression	65.0 (50.0–73.0)	1,180			0.384 ± 0.078		
Atteritano	2013	Italy	no depression	53.36 ± 2.47	50	0.82 ± 0.09	0.71 ± 0.07	*N*/A	0.66 ± 0.09	*N*/A
			depression	53.63 ± 1.93	50	0.72 ± 0.06	0.58 ± 0.04		0.54 ± 0.06	
Fazeli	2013	USA	no depression	<17	33	*N*/A	*N*/A	0.89 ± 0.14	0.96 ± 0.14	
			depression	<17	32			0.88±0.014	0.95 ± 0.15	
Calarge	2014	USA	no depression	19.1(1.4)	150	0.83 ± 0.89	*N*/A	*N*/A	*N*/A	*N*/A
			depression	19.1(1.4)	72	0.58 ± 1.02				
Rauma	2015	Finland	no depression	60.9 (47.6–75.1)	794	1.298 ± 0.201	*N*/A	0.418 ± 0.064	1.062 ± 0.147	*N*/A
			depression	53.5 (38.3–64.1)	144	1.263 ± 0.178		0.423 ± 0.064	1.083 ± 0.161	

### 3.2 Meta‐analyses results

#### 3.2.1 Overall meta‐analyses for BMD in MDD

**FIGURE 2:**
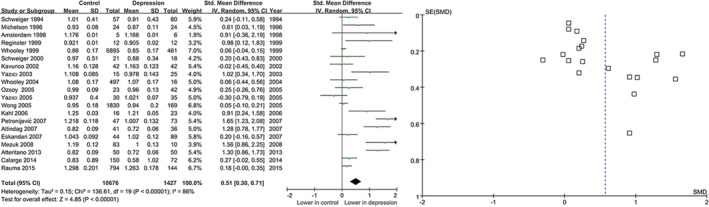
Forest for the summary effect size in the spine

Among the 26 included published studies, 20 studies examined the spine BMD in subjects with depression and controls. The result shows that subjects with MDD had a lower BMD than controls (STD=0.51, 95% CI =0.30–0.71, *p* <.00001) **(**Figure 2 **)**. There was a marked heterogeneity in spine BMD comparisons (I^2^=89%, Tau^2^=0.00, *p* <.00001). Then, 11 case–control studies, including 451 patients with MDD and 344 healthy controls, were pooled together to evaluate the relationship between MDD and BMD in the femoral neck.

**FIGURE 3:**
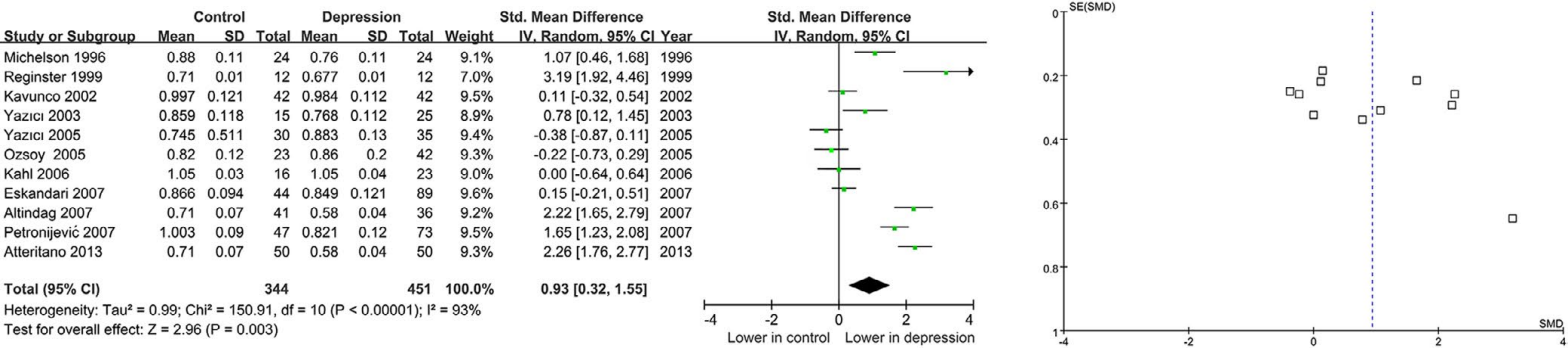
Forest plots for the summary effect size in the femoral neck

On the basis of the random‐effects model, the STD for BMD showed a significant correlation with lower bone mass under femoral neck (STD=0.93, 95% CI=0.32 to 1.55, *p* =.003) **(**Figure 3 **)**. There was a remarkable heterogeneity in spine BMD comparisons (I^2^=93%, Tau^2^=0.99, *p* <.00001).

**FIGURE 4:**
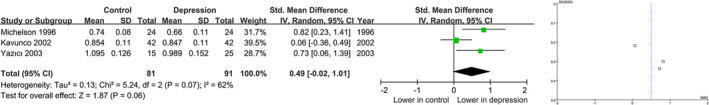
Forest plots for the summary effect size in the femoral trochanter

We also examined the femoral trochanter BMD in subjects with MDD and controls composed of 3 studies and observed that there is no significant difference of BMD under the femoral trochanter (STD=0.49, 95% CI=−0.02 to 1.01, *p* =.06) between depression and controls **(**Figure 4 **)**. Moderate heterogeneity was found in femoral trochanter BMD comparisons (I^2^=62%, Tau^2^=0.13, *p* =.07).

**FIGURE 5:**
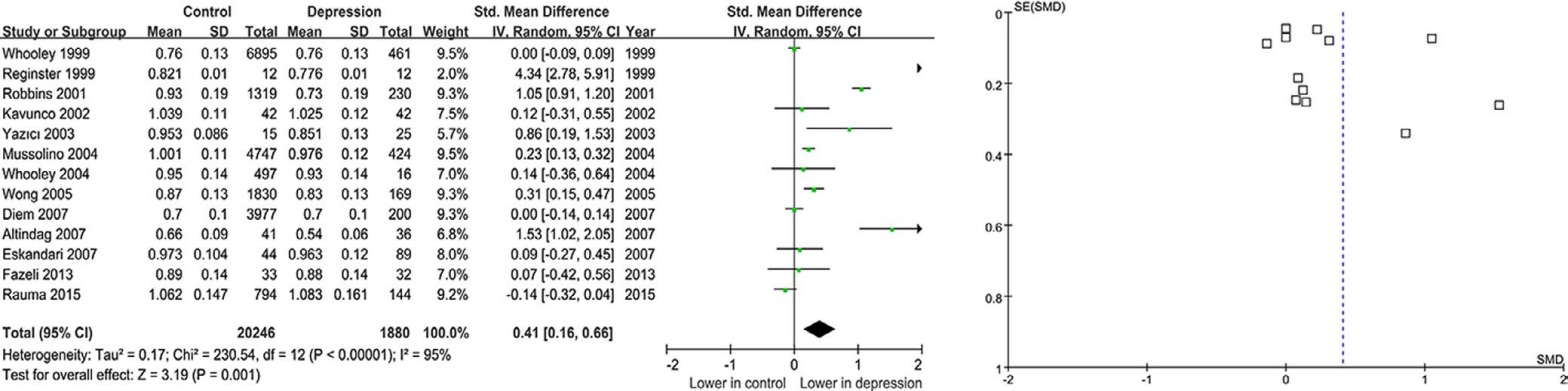
Forest plots the summary effect size in the hip

**FIGURE 6:**
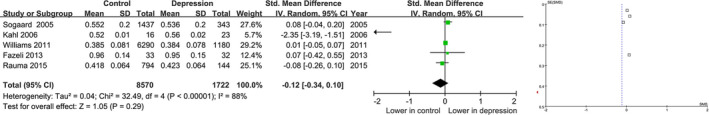
Forest plots for the summary effect size in the forearm

In the hip comparisons, the STD value was 0.41(95% CI=0.16 to 0.66, p =.001) by comparing the BMD between depression and controls, suggesting that the BMD was lower in depression**(**Figure 5 **)**. There was a remarkable heterogeneity in hip BMD comparisons (I^2^=95%, Tau^2^=0.17, *p* <.00001). However, no relationship between BMD and MDD was found under forearms BMD with STD‐0.12 (95% CI=−0.34 to 0.10, *p* =.29) **(**Figure 6 **)**.

#### 3.2.2 Subgroup and heterogeneity analysis

There was a remarkable heterogeneity among STDs in overall comparisons, and the subgroup analysis was carried out based on mean age, gender, recruitment diagnostic criteria, and measuring methods of all included studies. The characteristic of included studies is displayed in Table 2.

**TABLE 2:** Characteristic of Included Studies for Subgroup analysis

Study	Country	Group	Age	Measuring methods	Gender		Recruitment	Diagnostic criteria	Index
					F	M			
Schweiger	Germany	No depression	60(12)	Single energy quantitative CT	27	30	Clinical samples	DSM‐III‐R	BMD
		Depression	60.5(10.5)		53	27			
Michelson	USA	No depression	41(7)	Dual‐energy X‐ray	24	0	Clinical samples	DSM‐III‐R	BMD
		Depression	41(8)		24	0			
Amsterdam	USA	No depression	37.8(3.6)	Dual‐energy X‐ray	3	2	Clinical samples	DSM‐III‐R	BMD
		Depression	41.3(12.8)		4	2			
Reginster	Belgium	No depression	——	Dual‐energy X‐ray	12	0	Population based	Self‐rating (GHQ−28)	BMD
		Depression	——		12	0			
Whooley	USA	No depression	73.3(5.1)	Dual‐energy X‐ray	‐	‐	Population based	Self‐rating (GDS)	BMD
		Depression	74.5(5.3)		‐	‐			
Schweiger	Germany	No depression	64(10)	Single energy quantitative CT	7	14	Clinical samples	DSM‐III‐R	BMD
		Depression	59(11)		8	10			
Robbins	USA	No depression	74.21 (4.61)	Dual‐energy X‐ray	‐	‐	Population based	Self‐rating (CES‐Dm)	BMD
		Depression	74.87 (5.56)		‐	‐			
Kavunco	Turkey	No depression	36.7(6.7)	Dual‐energy X‐ray	42	0	Clinical samples	DSM‐IV	BMD
		Depression	35.4(7.5)		42	0			
Yazıcı	Turkey	No depression	31.2(7.9)	Dual‐energy X‐ray	15	0	Clinical samples	DSM‐IV	BMD
		Depression	30.8(8.4)		25	0			
Mussolino	USA	No depression	29.8	Dual‐energy X‐ray	‐	‐	Population based	Self‐rating (DIS)	BMD
		Depression	30.3		‐	‐			
Whooley	USA	No depression	66.7 ( 7.5)	Dual‐energy X‐ray	0	16	Population based	Self‐rating (GDS)	BMD
		Depression	64.6 ( 8.6)		0	497			
Ozsoy	Turkey	no depression	33.73 ± 7.16	Dual‐energy X‐ray	12	11	Clinical samples	DSM‐IV	BMD, Z‐score, T‐score
		depression	37.57 ± 8.70		21	21			
Sogaard	Norway	no depression	40.5	Dual‐energy X‐ray	1,437	‐	Population based	Self‐‐rating (custom)	BMD
		depression	40.7		343	‐			
Wong	Hongkong	no depression	72.34 ± 4.96	Dual‐energy X‐ray	0	1,830	Population based	Self‐rating (GDS)	BMD
		depression	72.94 ± 5.41		0	169			
Yazıcı	Turkey	no depression	46.2 ± 4.2	Dual‐energy X‐ray	30	0	Clinical samples	DSM‐IV	BMD, T‐score
		depression	44.8 ± 5.4		35	0			
Kahl	Germany	no depression	18–43	Dual‐energy X‐ray	16	0	Clinical samples	DSM‐IV	BMD, T‐score
		depression	20–51		23	0			
Altindag	Turkey	no depression	42.8 (5.3 )	Dual‐energy X‐ray	41	0	Clinical samples	DSM‐IV	BMD
		depression	39.8 (8.8)		36	0			
Diem	USA	no depression	75.6 ± 4.1	Dual‐energy X‐ray	3,977	0	Population based	Self‐rating (GDS)	BMD
		depression	76.7 ± 4.3		200	0			
Eskandari	USA	no depression	35 ± 6.8	Dual‐energy X‐ray	44	0	Clinical samples	DSM‐IV	BMD
		depression	35 ± 6.9		89	0			
Petronijević	Serbia	no depression	40.5 ± 5.7	Dual‐energy X‐ray	47	0	Clinical samples	DSM‐IV	BMD
		depression	40.7 ± 4.6		73	0			
Mezuk	USA	no depression	——	Dual‐energy X‐ray	55	28	Population based	Self‐rating(DIS)	BMD
		depression	——		7	3			
Williams	Australia	no depression	66.0	Dual‐energy X‐ray	‐	‐	Population based	Self‐rating	BMD
		depression	65.0		‐	‐			
Atteritano	Italy	no depression	53.36 ± 2.47	Dual‐energy X‐ray	50	0	Clinical samples	DSM‐IV	BMD, Z‐score, T‐score
		depression	53.63 ± 1.93		50	0			
Fazeli	USA	no depression	<17	Dual‐energy X‐ray	16	16	Clinical samples	DSM‐IV	BMD, Z‐score
		depression	<17		17	16			
Calarge	USA	no depression	19.1(1.4)	Dual‐energy X‐ray	43	29	Clinical samples	DSM‐IV	BMD, Z‐score
		depression	19.1(1.4)		110	40			
Rauma	Finland	no depression	60.9	Dual‐energy X‐ray	0	794	Population based	Self‐rating	BMD
		depression	53.5		0	144			

Results of subgroup analysis of BMD alteration in subjects of different ages are shown in Table 3. It was suggested that all the STDs, 95% CI, and *P* values were calculated and the significant heterogeneity remained.

**TABLE 3:** Subgroup analysis of BMD alteration in subjects of different age

	Spine		Total hip		Femoral neck		Forearm		Trochanter	
	STD (95% CI)	*P*	STD (95% CI)	*P*	STD (95% CI)	*P*	STD(95% CI)	*P*	STD (95% CI)	*P*
**Mean Age**										
Old age	0.09(0.02,0.16)	0.02	0.23(−0.14,0.60)	0.23	‐	‐	0.00(−0.06,0.06)	0.93	‐	‐
Adults	0.66(0.51, 0.81)	0.0009	0.44(0.08,0.80)	0.02	0.76(0.15,1.38)	0.02	−1.10(−3.48,1.28)	0.37	0.49(−0.02,1.01)	0.06
Adolescence	0.27(−0.02,0.55)	0.06	‐	‐			0.07(−0.42, 0.55)	0.78		
**Gender**										
Women	0.05 (0.00, 0.09)	0.05	0.04 (0.01, 0.07)	0.01	0.06 (0.02, 0.10)	0.002	−0.01 (−0.06, 0.03)	0.60	0.49(−0.02,1.01)	0.06
Men	0.07 (−0.02, 0.15)	0.12	0.02 (−0.03, 0.06)	0.45	‐	‐	−0.00 (−0.02, 0.01)	0.41	‐	‐
**Recruitment**										
Population based	0.26(0.08,0.45)	0.006	0.35 (0.05, 0.66)	0.02	‐	‐	0.02 (−0.03, 0.07)	0.50	‐	‐
Clinical samples	0.59(0.27,0.90)	0.0003	0.70 (0.10, 1.29)	0.02	0.76 (0.15, 1.38)	0.02	−1.11 (−3.48, 1.25)	0.36	0.49(−0.02,1.01)	0.06
**Diagnostic criteria**										
Self‐rating	0.26(0.08,0.45)	0.006	0.35 (0.05, 0.66)	0.02	‐	‐	0.02 (−0.03, 0.07)	0.50	‐	‐
Diagnostic interviews	0.59(0.27,0.90)	0.0003	0.70 (0.10, 1.29)	0.02	0.76 (0.15, 1.38)	0.02	−1.11 (−3.48, 1.25)	0.36	0.49(−0.02,1.01)	0.06
**Measuring methods**										
CT	0.23 (−0.07, 0.53)	0.14	‐	‐	‐		‐	‐	‐	‐
DEXA	0.52 (0.31, 0.73)	<0.00001	0.49 (0.24, 0.75)	0.0001	0.93 (0.32, 1.55)	0.003	−0.12 (−0.34, 0.10)	0.29	0.49(−0.02,1.01)	0.06

In terms of age, the subgroup was stratified into old age (>55 years), adult age (20–55 years), and adolescence (<20 years). The age‐stratified analysis indicated that lower BMD was greatly related to MDD in patients with depression under adult age at spine site, as well as the total hip and femoral neck. However, there was no correlation between BMD and depression at total hip in subjects under old age. Meanwhile, there was no significant difference of BMD at forearm and femoral trochanter between depression and controls at any age stage.

Gender stratification analysis showed that MDD was closely related to lower BMD risk in the female under spine, femoral neck, and total hip, but not in forearm and trochanter. However, there no relationship between lower hip BMD and MDD was found in male population among four studies with STD 0.02 (95% CI=−0.03 to 0.06, *p* =.45).

Recruitment and diagnostic criteria were performed and diagnosed based on self‐rating questionnaires (SR), and the retained studies were carried out with clinical samples using standard diagnostic criteria. Hence, the results in these two subgroups were analyzed to be same. Lower BMD kept still related to MDD in the depressive population under spine site, total hip, and femoral neck; but not in forearm and femoral trochanter (Table 3 **)**.

Additionally, two methods were used for BMD measuring, dual‐energy X‐ray (DEXA) and single energy quantitative CT. The latter one was only used in two studies both performed by Schweiger for spine BMD examination. Compared with CT method, lower BMD was suggested to be still related to MDD in the depressive population in spine site, total hip, and femoral neck using DEXA **(**Table 3 **)**.

#### 3.2.3 Sensitivity analysis and publication bias

Sensitivity analyses were carried out by the leave‐one‐out method to evaluate the degree that individual study affected the outcomes of the overall analysis. Sensitivity analysis indicated that no single study affected the pooled STDs. Egger's test suggested that there was no strong statistical evidence for publication bias (all *p* >.05).

## 4 DISCUSSION

Usually, areal BMD (g/cm^2^) was measured at the physical activity‐related sites including forearm, lumbar spine, total hip (the femoral neck, trochanter, Ward's triangle) using DEXA absorptiometry, and BMD was also a strong predictor of osteoporosis and fracture risk (Kalender et al. 1995; Kröger et al. 1995; Sievänen et al. 1992). Although large numbers of information have suggested that the depressive symptoms could be risk factors leading to osteoporosis and fracture in MDD. The association of major depression and osteoporosis was still a controversial issue due to study design and inclusion criteria. In our meta‐analysis, we analyzed the association of BMD and MDD under five common measured bone sites including spine, total hip, femoral neck, femoral trochanter, and forearm. Our findings showed that there is a significant decreased BMD in spine, total hip, and femoral neck. Meanwhile, according to the current meta‐analysis, compared with the control group, BMD of spine, femoral neck, and total femur of MDD patients decreased by 5.1%, 9.1%, and 4.1%, respectively. Nevertheless, there was no difference existed in forearm and femoral trochanter BMD between MDD and controls. Our results showed that MDD aggravated a risk of osteoporosis, and the sensitivity analysis further confirmed the stability of the results.

Additionally, several meta‐analyses have also found the relationship between MDD and osteoporosis or low BMD in case–control. Similarly, a synthesis meta‐analysis by Cizza et al. (2010) found a lower BMD at AP spine (4.73%), total femur (3.53%), and femoral neck (7.32%) than controls. Although there was no relationship between BMD and depression at forearm, BMD in the forearm should be paid more attentions due to that distal forearm was the most common site of fracture in childhood (Khosla et al. 2003), while the incidence of depression was increasing in the child and adolescence (Brown et al. 1999; Klerman, 1988). Moreover, physical activity is associated with BMD and depression, especially after weight‐bearing exercise, and low physical activity is associated with low BMD (Boot et al. 1997; Dalén & Olsson, 1974). In our meta‐analysis, weight‐bearing bones (spine, hip, and femoral neck) showed an increased risk to osteoporosis with lower BMD rather than non‐weight‐bearing bones (forearms) in MDD. Since physical activity has been able to prevent and decrease depressive symptoms, and higher levels of physical activity have been associated with lower depressive symptoms, forearms were always excised in common and may not be prone to getting bone mass loss as a result (Madsen et al. 1998).

As far as we know, multiple prospective studies have studied the association between BMD and depression in subjects of different age and carried out mostly in post‐menopausal women suggesting that the increased risk for fractures associates with increasing age for the same level of BMD (Atteritano et al. 2013; Aydin et al. 2011; Erez et al. 2012). As Our meta‐analysis results show that the relationship between spine bone density decline and MDD in the elderly, adults, and adolescents is well defined. However, it is worth noting that adult total hip bone density seems to be lower than that of older adults. The relationship between bone density and depression has been confirmed in adult women and men, but not in the elderly. The reason for the decreased bone density in adults and adolescents with depressive symptoms may be caused by several factors. Individuals with depressive symptoms have higher cortisol levels than healthy individuals, and cortisol is a potential mediator of BMD decline in adult depressed women (Altindag et al. 2007; Furlan et al. 2005). Poor eating habits and depressive lifestyles are also common in patients with depression, and diet and exercise are important factors in maintaining bone mass. Importantly, obesity has a negative effect on bones and has been shown to be associated with depression in adolescents and adults (Hirota et al. 1992; Tucker et al. 2002).

The present meta‐analysis clearly has indicated that assessment of an association between depression and BMD critically depends on the gender difference. The finding indicated that MDD which could decrease BMD was substantial in the female population but not in the male in gender‐stratified analysis. Multiple factors could be possible reasons for this difference between female and male. As known to all, women were prone to get depressed than men with a ratio 2:1, especially for post‐menopausal women (Areias et al. 1996; Kendler & Prescott, 1999). Hormonal factors such as levels of estrogen may affect the association of BMD and depression between men and women (Bone et al. 2000; Khosla et al. 1998; Kobayashi et al. 1996). Most of our included studies involved participants were aged women under menopausal status, which may affect depression as well as BMD in women.

There were also few disadvantages in our meta‐analyses. First of all, the sample size was limited by the numbers of included studies. The sample size was not enough for a comprehensive analysis between BMD and depression in femoral trochanter and forearm sites. In addition, the number of included samples was limited for the adolescence spine analysis, the forearm, and trochanter analysis. Therefore, further studies were needed to investigate the association between BMD and depression in the femoral trochanter and forearm sites. Second, English studies were included in the meta‐analysis, which were not sufficiently enough for excluding small study bias. Third, adult patients aged from 20 to 55 were included which might increase heterogeneity. Finally, although T or Z scores were also calculated as bone markers, we only analyzed the relationship within BMD and MDD because of the limited numbers of reported T or Z scores (four T scores, four Z scores) and the normalized methods for T or Z scores. Notably, the T or Z scores were all found to be related with depression in these studies.

## 5 CONCLUSION

In summary, this was an updated meta‐analysis to reveal the association between BMD and MDD in different bone sites. We found a strong and clinically significant association between MDD and low bone mass at spine, total hip, femoral neck, but not in forearm and femoral trochanter. What's more, adults and women appeared to have lower bone mineral density under depression. Our meta‐analysis may provide clinicians and public health administrators with an important screening tool for assessing depression and avoiding osteoporosis in adult subjects and female. Since many factors are related to bone mineral density, other factors (such as gender, age, and ethnicity) should be considered in future.

## CONFLICTS OF INTEREST

There are no potential conflicts of interest to disclose.

## ETHICAL APPROVAL

The study was approved by the Yongchuan Hospital, Chongqing Medical University. Informed consent was obtained. A statement that the study conforms to the Declaration of Helsinki, USA, and/or European Medicines Agency Guidelines for human subjects.

## Data Availability

Not applicable.
